# Glucose tolerance in a rural population of Bangladesh

**DOI:** 10.4103/0973-3930.43098

**Published:** 2008

**Authors:** M. A. Rahim, A. K. Azad Khan, S. M. K. Ali, Q. Nahar, A. Shaheen, A. Hussain

**Affiliations:** Institute of General Practice and Community Medicine, Department of International Health, University of Oslo, Norway; 1 Department of Epidemiology and Biostatistics, Research Division, Bangladesh Institute of Research and Rehabilitation in Diabetes, Endocrine and Metabolic Disorders (BIRDEM), Dhaka, Bangladesh; 2 Institute of Nutrition and Food Science, University of Dhaka, Dhaka, Bangladesh; 3 Center on the Family, University of Hawaii at Manoa, Honolulu, Hawaii, USA; 4 Institute of Psychiatric Research University of Oslo, Norway

**Keywords:** Body mass index, fasting blood glucose, glucose intolerance, rural Bangladesh

## Abstract

**CONTEXT:**

The prevalence of type 2 diabetes is increasing in the Bangladeshi population. However, there is little information available on the prevalence of glucose intolerance, ie, type 2 diabetes, impaired glucose tolerance, and impaired fasting glucose.

**AIMS:**

The main aim of this study is to determine the prevalence of different categories of glucose intolerance and their relationship with different anthropometric and demographic characteristics.

**SETTINGS AND DESIGN:**

This cross-sectional study was performed in a rural area of Bangladesh.

**MATERIALS AND METHODS:**

A random sample of 5000 persons aged ≥ 20 years was included in this study. Fasting blood glucose was measured in 3981 individuals and 2-h post-glucose blood glucose was measured in 3954 subjects after the known cases of diabetes (n = 27) were excluded. Height, weight, waist and hip circumference, and blood pressure were measured.

**STATISTICAL ANALYSIS:**

Pearson Chi-squared test and correlation test were used for analysis as appropriate.

**RESULTS:**

The prevalence of impaired fasting glucose (IFG), impaired glucose tolerance (IGT) and diabetes (DM) were 1.3, 2.0, and 7.0%, respectively. IFG, IGT, and IFG + IGT were more prevalent in females. Age showed a significant positive relationship with increasing levels of glucose intolerance. Body mass index, waist circumference, and waist-to-hip ratio were higher in the glucose-intolerant group than in the normal glucose tolerance (NGT) group. There was a positive correlation between FBG and 2-h BG in NGT and DM subjects.

**CONCLUSION:**

The FBG value identified more people with glucose intolerance than the 2-h BG. These findings will help developing diabetes preventive strategy in rural populations.

## Introduction

The prevalence of type 2 diabetes mellitus (T2DM) is projected to increase radically during the forthcoming decades in both developed and developing countries.[1] However, the prevalence of various stages of glucose intolerance, ie, T2DM, impaired fasting glucose (IFG) and impaired glucose tolerance (IGT), differ noticeably between countries and populations. It has been suggested that differential prevalence in the stages of glucose intolerance is related to the diet and genetic susceptibility of individuals, the age structure of the population, and the stage of economic development and level of urbanization of a country.[2] Bangladesh is a developing country which has experienced rapid urbanization, rural to urban migration, and increase in employment with limited economic development.[3] At the same time, the county has experienced a demographic transition with a slow but gradual increase in the proportion of the older age-groups.[4]

Some population-based studies conducted in urban and rural areas of Bangladesh have attempted to estimate the prevalence of diabetes mellitus. These studies, conducted at different time points, show a trend for increase in the prevalence of diabetes; the prevalence rates in these studies have ranged from 2.2 to 8.1% both in rural and urban communities.[5–7] A high prevalence of diabetes and chronic heart disease (CHD) has also been reported among Bangladeshis settled in the UK as compared to the native population.[8] Some other recent studies have shown a high prevalence of diabetes, IGT, and IFG in native Indians and in Asian Indians settled in Europe and in the USA.[9–11] IFG or IGT is now considered as pre-diabetes and is suggested to be a strong risk factor for cardiovascular disease (CVD).[12]

In Bangladesh, studies related to diabetes and IGT in urban areas have reported different prevalence rates.[6,13] The inconsistencies may have been due to variations in the sample sizes and the different methods used for blood glucose estimation. To the best of our knowledge, no study has yet been conducted in the Bangladeshi population to estimate the prevalence of glucose intolerance (T2DM, IFG, and IGT) using the American Diabetic Association (ADA) and World Health Organization (WHO) criteria.[14,15] Evidence suggests that in Pakistan, India, and Bangladesh, the risk factors for diabetes and CVD vary with the region being studied[16] The purpose of the present study was to use the new WHO - 1999 criteria to estimate the prevalence of different categories of glucose intolerance[15] and to identify the demographic and anthropometric characteristics of the sample population.

## Materials and Methods

This analysis was carried out with the data collected from a population-based cross- sectional survey conducted in 2004 in a rural community 40 km to the north of Dhaka city. The details of the study population have been described elsewhere.[17] In brief, a randomly selected population of 5,000 persons (both males and females; aged ≥20 years) were invited to participate in the study. Fasting blood glucose (FBG) was estimated in 3981 subjects and 2-h PG BG (post-glucose blood-glucose) was estimated in 3954 individuals; known cases of diabetes (n= 27) were excluded.

Weight, height, waist and hip circumferences were measured with the study participants in a standing position, wearing light clothes and no shoes. The weight was measured to the nearest 0.1 kg by a modern digital bathroom scale and the height was measured to the nearest 0.1 cm. Body mass index (BMI) was calculated by dividing the weight (in kilograms) by the height in meters squared. Waist circumference was measured at the level of the minimum circumference between the lower border of ribs and iliac crest. Hip circumference was measured at the greatest protrusions of the buttocks just below the iliac crest. The waist-hip ratio (WHR) was calculated as waist circumference / hip circumference.

Blood pressure (BP) was recorded after the subjects had rested for at least 5 min. The pressure was measured on the right arm using a mercury sphygmomanometer with a standard cuff for adults; the stethoscope bell was placed lightly over the brachial artery and the blood pressure was recorded to the nearest 2 mm Hg, reading from the top of the mercury meniscus. Systolic blood pressure (SBP) was recorded at the first appearance of Korotkoff sounds and diastolic blood pressure (DBP) was measured at phase V, ie, the disappearance of the sounds.

Capillary whole blood was taken for measurement of FBG, which was measured in all individuals (n = 3981) using a HemoCue glucose analyzer. The machine was calibrated every day with standard glucose solution to minimize the measuring error. After estimation of FBG, all participants (n = 3954), except the known diabetic cases, had a 2-h post-glucose BG estimation done.

Diabetes, IFG, and IGT were defined using the new WHO criteria 1999,[15] which are as follows:

**Table:** 

	FBG (mmol/l)		2-h post BG (mmol/l)
Normal (NGT)	< 5.5	and	< 7.7
Impaired fasting glucose	5.6–6.0	and	0.0< 7.7
Impaired glucose tolerance (IGT)	0.0< 5.5	and	7.8–11.0
*IFG + IGT	5.6–6.0	and	7.8–11.0
Diabetes	>6.1	and/or	>11.1

* Not mentioned in the WHO criteria

BMI > 25 kg/m^2^ was taken to indicate obesity for both sexes. The cut-off values for waist circumference for men and women were > 90 and > 80 cm, respectively and WHR for men was > 0.90 and > 0.85 for women.[18]

### Statistical analysis

The prevalence of diabetes, IFG, and IGT was determined by calculating the simple percentages. Comparisons between the groups was done using the χ^2^ test and Student's t test was used for continuous variables. Pearson correlation test was done to calculate the correlation between the variables. A *P*-value of < 0.05 was considered statistically significant. All *P*-values presented are two-tailed. We used SPSS, version 11.0, for analysis.

### Ethics

As most of the participants were illiterate, verbal consent was secured from each individual prior to inclusion in the study. They were also verbally informed of their right to withdraw from the study at any stage or to restrict their data from the analysis. The protocol was approved by both the Norwegian and Bangladeshi ethical committees for medical research.

## Results

The prevalence of IFG, IGT, IFG + IGT, and diabetes were 1.3, 2.0, 4.3, and 7.0%, respectively. IFG, IGT, and IFG + IGT were more prevalent in females than males, however the difference was significant only for IFG + IGT (5.0% in females vs 3.1% in males; *P* < 0.001) group. No significant sex difference was found in the diabetic or other groups. BMI was higher in the IGT, IFG + IGT, and DM groups, and increased waist circumference was observed in the IGT and DM groups [Table 1].

**Table 1:** Prevalence of different categories of glucose tolerance (by sex) and percentage of the sample with increased body mass index, waist circumference, and waist-hip ratio

	NGT		IFG		IGT		IFG + IGT		DM	
					
	No.	(%)	No.	(%)	No.	(%)	No.	(%)	No.	(%)
Total	3,387	(85.4)	53	(1.3)	79	(2.0)	169	(4.3)	279	(7.0)
Male	1,383	(86.9)	17	(1.1)	23	(1.4)	50	(3.1)***	119	(7.5)
Female	2,004	(84.2)	36	(1.5)	56	(2.4)	119	(5.0)***	160	(6.7)
Increased BMI	321	(9.5)	5	(9.4)	11	(14.1)	26	(15.2)	60	(21.5)
Increased waist circumference	481	(14.2)	7	(13.2)	23	(29.1)	41	(24.0)	79	(28.3)
Increased WHR	1,950	(57.5)	33	(62.2)	55	(69.6)	113	(66.8)	203	(72.7)

NGT (FBG = 0.0–5.5 mmol/l and 2–h BG = 0.0–7.7 mmol/l), IFG (FBG = 5.6–6.0 mmol/l and 2–h BG = 0.0–7.7 mmol/l), IGT (FBG = 0.0–5.5 mmol/l and 2–h BG = 7.8–11.0 mmol/l), IFG + IGT (FBG = 5.6–6.0 mmol/l and 2–h BG = 7.8–11.0 mmol/l), DM (FBG ≥ 6.1 mmol/l or 2–h BG ≥ 11.0 mmol/l)

*** *P*<0.001

The mean age of the subjects increased with increasing degree of glucose intolerance in both sexes. BMI, WHR, and waist circumference were higher in the glucose intolerant groups than in the NGT group, while the highest mean values were found in the diabetes group for both sexes. WHR was significantly higher among males in all groups. Waist circumference was significantly higher in males in the IFG and DM groups [Table 2].

**Table 2:** Mean ± 2SD of age, BMI, waist, WHR, SBP, and DBP by different categories of glucose tolerance (males and females)

	NGT		IFG		IGT		IFG + IGT		DM	
					
	Mean	2SD	Mean	2SD	Mean	2SD	Mean	2SD	Mean	2SD
Age										
Male	37.9***	± 14.6	37.2	± 14.9	44.6	± 17.3	43.9	± 16.9	49.2***	± 16.1
Female	34.9***	± 12.9	41.3	± 19.7	40.4	± 17.0	41.2	± 15.3	42.6***	± 14.8
BMI (kg/m^2^)										
Male	20.4^*^	± 2.9	20.8	± 2.6	20.7	± 3.0	20.7	± 3.2	22.1	± 4.2
Female	20.7^*^	± 3.3	20.0	± 3.7	20.6	± 3.6	21.0	± 4.3	21.3	± 4.6
Waist (cm)										
Male	73.8***	± 8.5	76.6^*^	± 9.2	75.2	± 11.7	75.9	± 9.7	79.6^*^	± 11.6
Female	72.0***	± 9.1	70.0^*^	± 9.5	72.9	± 10.6	73.9	± 11.4	75.3^*^	± 10.4
WHR										
Male	0.88***	± 0.06	0.89	± 0.05	0.88^*^	± 0.09	0.89***	± 0.07	0.92***	± 0.07
Female	0.83***	± 0.06	0.83	± 0.05	0.85^*^	± 0.07	0.85***	± 0.06	0.86***	± 0.07
SBP (mm Hg)										
Male	118.6	± 15.4	123.5	± 18.5	128.2	± 21.6	124.9	± 20.9	129.7	± 22.0
Female	118.4	± 17.5	121.6	± 18.7	123.4	± 20.5	123.6	± 20.3	127.8	± 21.8
DBP (mm Hg)										
Male	76.6	± 9.9	80.8	± 10.4	81.0	± 10.4	80.7	± 12.5	82.9	± 11.6
Female	76.3	± 11.1	78.6	± 10.1	79.1	± 13.4	79.8	± 10.5	80.9	± 11.9

*** *P* <0.001

Age has a significant positive relationship with the degree of glucose intolerance and this is observed in both males and females [Figure 1A and B].

**Figure 1A:**
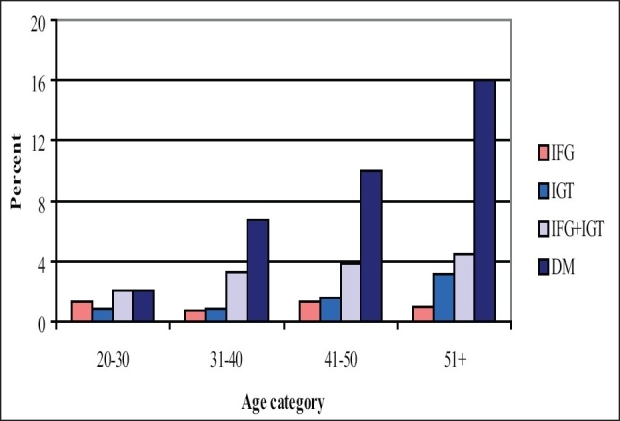
Prevalence of different categories of glucose intolerance by age (males)

**Figure 1B:**
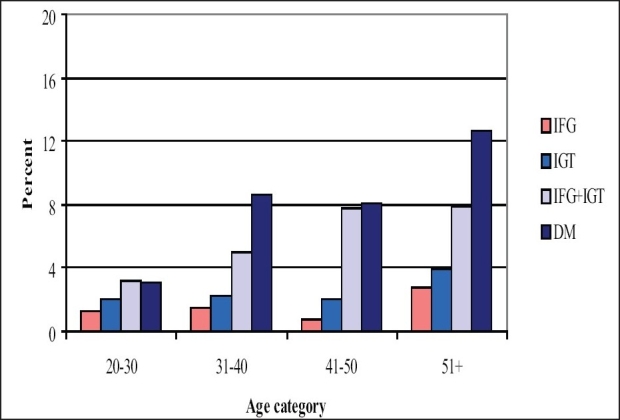
Prevalence of different categories of glucose intolerance by age (females)

Among the subjects with FBG value in the range of 5.6–6.0 mmol/l, 23.2% turned out to have NGT, 74.1% had IGT, and 2.6% were diabetic as assessed by the 2-h post-glucose BG values. Among the 258 diabetic subjects having an FBG of ≥ 6.1 mmol/l, 128 (49.6%) had 2-h PG BG ≥11.1 mmol/l and 44.6% had 2-h PG BG level in the range of 7.8–11.0 mmol/l. Among the total of 266 subjects with diabetes, 128 subjects had 2-h PG BG values of ≥_11.1 mmol/l and the remaining 130 subjects satisfied only the FBG criterion for diagnosis of diabetes (ie, FBG ≥ 6.1 mmol/l) [Table 3].

**Table 3:** Distribution of subjects according to fasting blood glucose and 2–h post-glucose BG categories

FBG (mmol/l)	2–h Post-glucose blood glucose (mmol/l)							
	
	NGT (0.0–7.7)		IGT (7.8–11.00)		DM (11.1+)		Total	
				
	No.	%	No.	%	No.	%	No.	%
NGT (0.0–5.5)	3,387	97.7	79	2.3	2	0.1	3,468	87.7
IFG (5.6–6.0)	53	23.2	169	74.1	6	2.6	228	5.7
DM (6.1+)	16	6.2	114	44.6	128	49.6	258	6.7
Total	3,456	87.4	362	9.2	136	3.4	3,954	100

FBG and 2-h post-glucose BG values were positively correlated in NGT and DM subjects. The correlation (Pearson) values and significance levels between FBG and 2-h post-glucose BG values for NGT and DM for males, females, and all subjects are presented in Table 4.

**Table 4:** Mean values of fasting blood glucose and corresponding 2–h post-glucose BG for different categories of glucose tolerance (male and female)

Mean value	NGT		IFG		IGT		IFG + IGT		DM	
					
(mmol/l)	Male	Female	Male	Female	Male	Female	Male	Female	Male	Female
FBG	4.4**	4.5**	5.7	5.7	4.7*	5.0*	5.8**	5.7**	8.1	8.0
2-h BG	4.9***	5.4***	5.0**	6.1**	8.5	8.3	8.5	8.5	12.4	12.6
n	1,383	2,004	17	36	23	56	50	119	114	152

* *P* < 0.05;

** *P* < 0.01;

*** *P* < 0.001;

Note: *P* values indicate significance of the difference in mean values of FBG and 2-h BG by gender among different groups of glucose tolerance.

## Discussion

Our data showed that the prevalence of IGT was significantly higher than that of IFG. The prevalence of IGT was slightly higher in females but no such sex difference was observed for IFG. Female subjects showed significantly higher prevalence of IFG + IGT, whereas the prevalence of diabetes did not vary between male and female subjects. However, earlier data has shown a higher prevalence of diabetes among female subjects.[19] The prevalence of IFG and IGT was found to vary in terms of its ascendancy across studies. IGT was found to be more prevalent compared to IFG in Mauritius,[20] USA,[21] and in Pima Indians.[22] However, studies conducted in the Netherlands,[23] Finland,[24] India,[9] and among Asian Indians[11] did not find any difference in the prevalence of IFG and IGT. Our results showed similar levels of IFG and IGT in men; however, IGT was higher in women compared to IFG, and this finding is consistent with previous studies in Bangladesh.[7,13] A sex difference in the prevalence of IGT has been reported in other countries.[25] The prevalence of IGT was found to be similar in both sexes in an urban Indian study.[26] Some other studies have shown a higher prevalence of IFG among men and a higher prevalence of IGT among women.[21,27]

Adverse anthropometric features are more prevalent among subjects having impaired glucose regulation, ie, those with IFG, IGT, IFG + IGT, and DM. Our data suggests that IGT and DM were more common among older participants. A similar finding on IFG was also observed in India.[11] Contrary to this finding, a report from Austria showed that age-related increase in FBG was particularly seen in subjects with IFG.[28] This difference between the findings of our study and that from India as compared to the studies conducted elsewhere are likely due to ethnic differences.

Our data showed that when FBG > 6.1 mmol/l was used as the diagnostic criterion, the prevalence of diabetes was 6.7%, whereas it was 3.4% when the 2-h post glucose BG values were used. This finding is an agreement with an Indian study where the prevalence of diabetes was 5.2% according to the FBG criteria and 4.3% according to 2-h post-glucose BG threshold.[29] Furthermore, our data showed that the agreement between the two procedures (FBG and 2-h post-glucose BG) for the identification of diabetic cases was around 50%. Concern has been expressed that IFG might not identify the same subjects as IGT.[29] However, the prevalence of glucose intolerance by either procedure was similar [(by FBG (IFG + DM) was 12.4% or 2-h post-glucose BG (IGT + DM)] was 12,6%)).

## Conclusion

The prevalence of glucose intolerance varied significantly, depending on the diagnostic criterion used, namely, FBG or 2-h post-glucose BG estimates. However, it is very important to detect the larger number of subjects with glucose intolerance to prevent diabetes. Our findings need to be verified in other settings for the development of an intervention strategy for the prevention and early management of abnormal glucose tolerance.
